# Frauenförderung in der Urologie am Beispiel der Habilitation

**DOI:** 10.1007/s00120-022-01915-3

**Published:** 2022-07-28

**Authors:** Maria-Noemi Welte, Sophie Knipper, Carolin Siech, Eva Maria Greiser, Laura Wiemer, Karina Müller, Laura Bellut, Margarete Teresa Walach, Annika Herlemann, Sarah Weinberger

**Affiliations:** 1 Klinik für Urologie, Universitätsklinikum Frankfurt, Frankfurt am Main, Deutschland; 2 Martini-Klinik Prostatakarzinomzentrum, Universitätsklinikum Hamburg-Eppendorf, Hamburg, Deutschland; 3 Mathias-Spital, Klinikum Rheine, Rheine, Deutschland; 4 Pro Uro Berlin, Berlin, Deutschland; 5 Urologische Klinik, Klinikum Bremen Mitte, Bremen, Deutschland; 6 Klinik für Urologie und Kinderurologie, Universitätsklinikum Erlangen, Erlangen, Deutschland; 7 Klinik für Urologie und Urochirurgie, Universitätsmedizin Mannheim, Mannheim, Deutschland; 8 Urologische Klinik und Poliklinik, Campus Großhadern, LMU München, München, Deutschland; 9 Urologische Klinik, Charité – Universitätsmedizin Berlin, Berlin, Deutschland

**Keywords:** Urologinnen, Karriere, Forschungsförderung, Genderaspekte, Mentoring, Female urologist, Career, Research funding, Gender issues, Mentoring

## Abstract

**Hintergrund:**

Der Anteil an Ärztinnen im Fachgebiet Urologie nimmt stetig zu. Dabei ist die zunehmende Feminisierung in Führungspositionen noch nicht angekommen. Eine Habilitation, die die Grundlage für eine wissenschaftlich erfolgreiche Karriere ist, wird von Urologinnen in Deutschland deutlich seltener als von ihren männlichen Kollegen erreicht.

**Ziel der Arbeit:**

Um Faktoren für eine effektivere Förderung von Frauen in der Urologie zu identifizieren, wurden die aktuelle Situation sowie Einflussfaktoren auf eine erfolgreiche Habilitation von Frauen in der Deutschen Gesellschaft für Urologie e. V. (DGU) untersucht.

**Methodik:**

Ein Online-Fragebogen wurde an 1343 weibliche Mitglieder der DGU in Deutschland, Österreich und der Schweiz verschickt. Die Antworten von 521 Ärztinnen wurden bezüglich Basischarakteristika und in Bezug auf Forschungsförderung statistisch ausgewertet. Der primäre Endpunkt der vorliegenden Arbeit war die Habilitationsrate.

**Ergebnisse:**

Insgesamt wurde der Fragebogen von 521 in der Urologie beschäftigten Ärztinnen zwischen 25 und 67 (Median 37) Jahren ausgefüllt. Hiervon waren die meisten Ärztinnen in Weiterbildung (*n* = 168, 32 %), arbeiteten in Vollzeit (*n* = 324, 62 %) und hatten Kinder (*n* = 277, 53 %). Insgesamt waren 359 (69 %) der Teilnehmerinnen promoviert und 63 (12 %) arbeiteten noch an ihrer Promotion. 30 (5,8 %) Urologinnen waren habilitiert. In univariablen logistischen Regressionsmodellen waren das Alter (Odds Ratio [OR]: 1,06), das Arbeitszeitmodell (Teilzeit OR: 0,19), ein Forschungsaufenthalt (OR: 21,4), eine Freistellung (OR: 13,7), die Teilnahme an einem Förderprogramm (OR: 6,9) oder Mentoring-Programm (OR: 7,0) unabhängige Prädiktoren für eine erreichte Habilitation. Ob eine Urologin Kinder hatte, war kein unabhängiger Prädiktor. In multivariablen logistischen Regressionsmodellen waren Alter (OR: 1,08) sowie ein Forschungsaufenthalt (OR: 9,04) unabhängige Prädiktoren für eine erreichte Habilitation.

**Schlussfolgerungen:**

Eine Förderung von Habilitationen unter Ärztinnen im Fachgebiet Urologie erscheint erstrebenswert, um den Frauenanteil in Führungspositionen zu steigern. Die Ergebnisse der Datenauswertung zeigen, dass insbesondere die Förderung von Forschungsaufenthalten explizit für Frauen ein vielversprechender Ansatz ist.

## Abstract (en)

**Background:**

The proportion of female urologists is steadily increasing, yet they continue to be underrepresented in academic leadership positions. A postdoctoral lecture qualification (habilitation), which is mandatory for a successful scientific career, is achieved significantly less often by female urologists in Germany than by their male colleagues.

**Objective:**

To identify factors to effectively promote female urologists into academic leadership positions, the current situation, as well as factors influencing successful habilitation of women who are members of the German Society of Urology (Deutsche Gesellschaft für Urologie [DGU]) were investigated.

**Methods:**

An online questionnaire was distributed to 1343 female members of the DGU in Germany, Austria, and Switzerland. The responses of 521 women were statistically analyzed with respect to baseline characteristics and in relation to research funding. The primary endpoint of our study was the habilitation rate.

**Results:**

The average age of the 521 participating female urologists who completed the questionnaire was 37 years (range 21–67 years). Of these, most female physicians were in postgraduate training (*n* = 168, 32%), worked full-time (*n* = 324, 62%), and had children (*n* = 277, 53%). Overall, 359 (69%) of the participants had a PhD and 63 (12%) were still working on their PhD. Thirty (5.8%) female urologists had a habilitation. In univariable logistic regression models, age (odds ratio [OR] 1.06), working time model (part-time OR 0.19), a research fellowship (OR 21.4), release from clinical work for research purposes (OR 13.7), and participation in a funding program (OR 6.9) or mentoring program (OR 7.0) were independent predictors of achieving habilitation. Whether a urologist had children was not an independent predictor of achieving habilitation. In multivariable logistic regression models, age (OR 1.08), and a research fellowship (OR 9.04) were independent predictors of achieving habilitation.

**Conclusions:**

Promoting habilitation among female urologists is required in order to increase the proportion of women in leading academic positions. The results of the data analysis show that the promotion of research fellowships explicitly for women could be a promising approach.

## Hintergrund und Fragestellung

Der Anteil an Ärztinnen im Fachgebiet Urologie nimmt stetig zu. Im Jahr 2008 lag die Frauenquote in der Urologie laut Ärztestatistik der Bundesärztekammer noch bei 11,3 %. Im Jahr 2021 waren von 6467 berufstätigen Urolog:innen 1335 weiblich, was einem Frauenanteil von 20,64 % und somit einem relativen Zuwachs von 82 % entspricht. Von den urologischen Ärzt:innen in Weiterbildung (ÄiW) waren 2021 55 % weiblich und die Frauenquote der Facharztanerkennungen stieg von 32,5 % im Jahr 2017 auf 38,3 % 2019 [1]. In Führungspositionen ist diese Entwicklung jedoch noch nicht angekommen. Dieser Beitrag befasst sich mit der Frage, wie eine Steigerung der Frauenquote in Führungspositionen erreicht werden kann.

Obwohl die Zahlen zeigen, dass die Urologie insgesamt weiblicher wird, sieht die Situation für Urologinnen in Führungspositionen anders aus. Dies wird durch die Erhebung „Medical Women on Top“ aus dem Jahr 2022 des Deutschen Ärztinnenbundes (DÄB) deutlich. Hiernach liegt der Anteil von Oberärztinnen aller Fachrichtungen in Deutschland bei 37 %, der Anteil an Oberärztinnen in der Urologie bei 30 %. Bei Führungspersonen bzw. Klinikdirektor:innen ist der Frauenanteil in Deutschland noch geringer. Lediglich 13 % aller Führungspositionen in der Humanmedizin werden durch Frauen besetzt. Tatsächlich wird dieser bereits niedrige Wert in der Urologie noch unterschritten. Mit nur zwei urologischen Klinikdirektorinnen in 36 Medizinischen Fakultäten liegt der prozentuale Anteil an weiblichen Führungskräften in Universitätskliniken bei 5 % [2]. Zudem zeigte eine Forschergruppe aus den Vereinigten Staaten 2017, dass obwohl die Anzahl der Urologinnen insgesamt steigt, Frauen weiterhin mit beruflicher Benachteiligung rechnen müssen. Frauen werden später als Männer befördert, erhalten geringere Forschungsgelder sowie durchschnittlich niedrigere Gehälter [3].

Nach einer Analyse der Deutschen Gesellschaft für Urologie e. V. (DGU) zum Status von Frauen an Universitätskliniken in Deutschland liegt der Frauenanteil bei 42,4 % unter ÄiW und 44 % der Fachärzt:innen, aber nur bei 25,3 % der Oberärzt:innen. Eine Abfrage der DGU-Mitgliederdatenbank im Mai 2022 ergab einen Frauenanteil von 24,8 % (1202/4843) unter deutschen DGU-Mitgliedern, während nur 18/167 (10,8 %) der Privatdozent:innen und 20/251 der Professor:innen (8,0 %) weiblich waren.

Um der zunehmenden Feminisierung in der Urologie gerecht zu werden, bedarf es somit einer gezielten strukturierten Frauenförderung. Dies hat sich die 2021 neu gegründete Arbeitsgemeinschaft (AG) Urologinnen zur Aufgabe gemacht. Voraussetzung für die Umsetzung effektiver Fördermaßnahmen einer wissenschaftlichen Karriere ist die Kenntnis der aktuellen Situation von Urologinnen, die in diesem Beitrag dargelegt werden soll.

## Material und Methoden

Im Rahmen der auf dem 71. DGU-Kongress gegründeten AG Urologinnen erstellte die Unterarbeitsgruppe „Bestandsaufnahme“ zwischen November 2021 und Februar 2022 einen Fragebogen zur Erhebung der aktuellen beruflichen Situation von Urologinnen in Deutschland, Österreich und der Schweiz. Der Link zum Online-Fragebogen wurde am 28.02.2022 über die E‑Mail-Verteilerliste der DGU an insgesamt 1343 weibliche Mitglieder der DGU verschickt und bis zum 30.03.2022 von insgesamt 521 Ärztinnen beantwortet.

### Fragebogen und Endpunkt

Von insgesamt 43 Fragen zu u. a. Alter, Berufstätigkeit, Zufriedenheit und familiärer Situation wurde in der vorliegenden Arbeit das Thema wissenschaftliche Tätigkeit untersucht. Hierbei wurden die Daten zu folgenden Fragen ausgewertet: „Sind Sie promoviert?“, „Sind Sie habilitiert?“, „Haben Sie einen Forschungsaufenthalt absolviert?“, „Sind oder waren Sie für Ihre Forschungsaktivität freigestellt?“, „Sind Sie durch ein Förderprogramm unterstützt worden?“ und „Haben Sie an einem Mentoring-Programm teilgenommen?“. Die Antworten auf diese 6 Fragen wurden insbesondere in Bezug auf Alter sowie familiäre Situation untersucht. Der primäre Endpunkt der vorliegenden Arbeit war die Habilitationsrate.

### Statistische Analysen

Zu den deskriptiven Statistiken gehörten Häufigkeiten und Proportionen für kategoriale Variablen. Für kontinuierlich kodierte Variablen wurden Mittelwerte, Mediane und Spannen angegeben. Die statistische Signifikanz von Unterschieden in den Medianen und Proportionen wurde mit den Kruskal-Wallis- und χ^2^-Tests bewertet. Univariable und multivariable logistische Regressionsmodelle untersuchten die Beziehung zwischen der Habilitationsrate und verschiedene Variablen wie Alter, familiäre Situation, Forschungsaufenthalte, Freistellung zur Forschungsaktivität, Teilnahme an Förderprogrammen oder Mentoring-Programmen. Die Prädiktoren wurden aus potenziellen Faktoren ausgewählt, die bereits zuvor als förderlich beschrieben wurden. Sie wurden in die multivariablen Modelle aufgenommen, wenn sie in der univariablen Analyse signifikant mit dem Ergebnis assoziiert waren. Für alle statistischen Analysen wurde die R‑Softwareumgebung für statistische Berechnungen und Grafiken (Version 3.4.3; R Foundation for Statistical Computing, Wien, Österreich) verwendet. Alle Tests waren zweiseitig und das Signifikanzniveau wurde auf *p* < 0,05 festgelegt.

## Ergebnisse

Insgesamt wurde der Fragebogen von 521 Ärztinnen zwischen 25 und 67 (Median 37) Jahre ausgefüllt. Hiervon waren die meisten Ärztinnen in Weiterbildung (*n* = 168, 32 %), arbeiteten in Vollzeit (*n* = 324, 62 %) und hatten Kinder (*n* = 277, 53 %). Insgesamt waren 359 (69 %) der Teilnehmerinnen promoviert und 63 (12 %) arbeiteten noch an ihrer Promotion. 30 (5,8 %) Urologinnen waren habilitiert (Tab. 1).

**Table 1:** 

Charakteristika	*n* = 521
*Wie alt sind Sie? (Jahre)*	37 (33, 45)^a^
*Wie ist ihr beruflicher Status?*	
Weiterbildung	168 (32 %)
Fachärztin	137 (26 %)
Oberärztin/leitende Ärztin	111 (21 %)
Chefärztin	6 (1,2 %)
Praxis (Angestellte, Teilhaberin, Inhaberin)	88 (17 %)
Keine Angabe	11 (2,1 %)
*In welchem Arbeitszeitmodell arbeiten Sie?*	
Vollzeit	324 (62 %)
Teilzeit	179 (34 %)
Derzeit nicht berufstätig	12 (2,3 %)
Keine Angabe	6 (1,2 %)
*Haben Sie Kinder?*	
Ja	277 (53 %)
Nein	240 (46 %)
Keine Angabe	4 (0,8 %)
*Sind Sie promoviert?*	
Ja	359 (69 %)
Nein	96 (18 %)
Nein, ich arbeite zur Zeit noch an meiner Promotion	63 (12 %)
Keine Angabe	3 (0,6 %)
*Sind Sie habilitiert?*	
Ja	30 (5,8 %)
Nein, ich arbeite zur Zeit noch an meiner Habilitation	55 (11 %)
Nein, ich strebe keine Habilitation an	423 (81 %)
Keine Angabe	13 (2,5 %)
*Haben Sie einen Forschungsaufenthalt absolviert?*	
Ja, im Ausland	34 (6,5 %)
Ja, im Inland	12 (2,3 %)
Nein	455 (87 %)
Keine Angabe	20 (3,8 %)
*Sind oder waren Sie für Ihre Forschungsaktivität freigestellt?*	
Ja	36 (6,9 %)
Nein	436 (84 %)
Keine Angabe	49 (9,4 %)
*Sind Sie durch ein Förderprogramm unterstützt worden?*	
Ja	18 (3,5 %)
Nein	432 (83 %)
Keine Angabe	71 (13 %)
*Haben Sie an einem Mentoring-Programm teilgenommen?*	
Ja	26 (5,0 %)
Nein	448 (86 %)
Keine Angabe	47 (9,0 %)

^a^Median (Interquartilsabstand); *n* (%)

Von den habilitierten (vs. nicht-habilitierte) Urologinnen hatten 57 % (vs. 6 %) einen Forschungsaufenthalt absolviert und 43 % (vs. 5 %) waren für eine Forschungsaktivität freigestellt worden. 13 % (vs. 3 %) der habilitierten (vs. nicht-habilitierte) Urologinnen waren durch ein Förderprogramm unterstützt worden und 20 % (vs. 4 %) hatten an einem Mentoring-Programm teilgenommen (Tab. 2).

**Table 2:** 

Charakteristika	Habilitation *n* = 30	Keine Habilitation *n* = 478
*Wie alt sind Sie? (Jahre)*	40 (37, 56)^a^	37 (33, 44)^a^
*Wie ist ihr beruflicher Status?*		
Weiterbildung	2 (6,7 %)	162 (34 %)
Fachärztin	5 (17 %)	130 (27 %)
Oberärztin/leitende Ärztin	16 (53 %)	94 (20 %)
Chefärztin	3 (10 %)	2 (0,4 %)
Praxis (Angestellte, Teilhaberin, Inhaberin)	1 (3,3 %)	85 (18 %)
Keine Angabe	3 (10 %)	5 (1,0 %)
*In welchem Arbeitszeitmodell arbeiten Sie?*		
Vollzeit	26 (87 %)	290 (61 %)
Teilzeit	3 (10 %)	173 (36 %)
Derzeit nicht berufstätig	0 (0 %)	12 (2,5 %)
Keine Angabe	1 (3,3 %)	3 (0,6 %)
*Haben Sie Kinder?*		
Ja	17 (57 %)	256 (54 %)
Nein	12 (40 %)	221 (46 %)
Keine Angabe	1 (3,3 %)	1(0,2 %)
*Haben Sie einen Forschungsaufenthalt absolviert?*		
Ja, im Ausland	12 (40 %)	22 (4,6 %)
Ja, im Inland	5 (17 %)	7 (1,5 %)
Nein	12 (40 %)	437 (91 %)
Keine Angabe	1 (3,3 %)	12 (2,5 %)
*Sind oder waren Sie für Ihre Forschungsaktivität freigestellt?*		
Ja	13 (43 %)	23 (4,8 %)
Nein	17 (57 %)	411 (86 %)
Keine Angabe	0	44 (9,2 %)
*Sind Sie durch ein Förderprogramm unterstützt worden?*		
Ja	4 (13 %)	14 (2,9 %)
Nein	17 (57 %)	408 (85,4)
Keine Angabe	9 (30 %)	56 (12 %)
*Haben Sie an einem Mentoring-Programm teilgenommen?*		
Ja	6 (20 %)	20 (4,2 %)
Nein	18 (60 %)	421 (88 %)
Keine Angabe	6 (20 %)	37 (7,8 %)

^a^Median (Interquartilsabstand); *n* (%)

In univariablen logistischen Regressionsmodellen waren das Alter (Odds Ratio [OR]: 1,06), das Arbeitszeitmodell (Teilzeit OR: 0,19), ein Forschungsaufenthalt (OR: 21,4), eine Freistellung (OR: 13,7), die Teilnahme an einem Förderprogramm (OR: 6,86) oder Mentoring-Programm (OR: 7,02) unabhängige Prädiktoren für eine erreichte Habilitation (Tab. 3). Ob eine Urologin Kinder hatte, war kein unabhängiger Prädiktor bzgl. der Habilitation.

**Table 3:** 

	Univariables logistisches Regressionsmodell				Multivariables logistisches Regressionsmodell			
Variable	OR	KI 2,5 %	KI 97,5 %	*p*-Wert	OR	KI 2,5 %	KI 97,5 %	*p*-Wert
*Wie alt sind Sie? (kontinuierlich kodiert)*	1,06	1,03	1,10	0,001	1,08	1,02	1,14	0,007
*In welchem Arbeitszeitmodell arbeiten Sie?*								
Vollzeit	Ref.				Ref.			
Teilzeit	0,19	0,04	0,56	0,008	0,20	0,03	0,87	0,06
*Haben Sie Kinder?*								
Nein	Ref.				–			
Ja	1,22	0,58	2,68	0,6	–			
*Haben Sie einen Forschungsaufenthalt absolviert?*								
Nein	Ref.				Ref.			
Ja	21,4	9,41	50,1	<0,001	9,04	2,12	35,9	0,002
*Sind Sie für Ihre Forschungsaktivität freigestellt worden?*								
Nein	Ref.				Ref.			
Ja	13,7	5,88	31,6	<0,001	4,12	0,58	32,7	0,2
*Haben Sie an einem Förderprogramm teilgenommen?*								
Nein	Ref.				Ref.			
Ja	6,86	1,80	21,6	0,002	0,39	0,04	2,79	0,4
*Haben Sie an einem Mentoring-Programm teilgenommen?*								
Nein	Ref.				Ref.			
Ja	7,02	2,34	18,8	<0,001	3,65	0,74	15,9	0,09

*OR* Odds Ratio, *KI* Konfidenzintervall, *Ref.* Referenz

In multivariablen logistischen Regressionsmodellen waren Alter (OR: 1,08) sowie ein Forschungsaufenthalt (OR: 9,04) unabhängige Prädiktoren für eine erreichte Habilitation (Tab. 3).

## Diskussion

Der Anteil an Ärztinnen im Fachgebiet Urologie nimmt stetig zu. Dies ist eine Entwicklung, die in Führungspositionen noch nicht angekommen ist. Die AG Urologinnen hat sich die Evaluation von Missständen und gezielten Fördermöglichkeiten von Ärztinnen in der Urologie zum Ziel gemacht.

In unserer Umfrage zum Status quo beantworteten 521 von 1343 Urologinnen den Fragebogen, was einer Rücklaufquote von 38,8 % der angeschriebenen Ärztinnen entspricht. Dies entspricht im Vergleich zu anderen Befragungen einer repräsentativen Quote [4].

Die in der Einleitung erwähnten Daten aus der DGU-Mitgliederdatenbank zeigen, dass die Habilitationsrate von Frauen in der Urologie in Deutschland weit hinter der ihrer männlichen Kollegen zurückliegt. Von den 3641 männlichen DGU-Mitgliedern sind 10,4 % habilitiert (149 Privatdozenten, 231 Professoren). Unter den 1202 weiblichen DGU-Mitgliedern sind hingegen nur 3,2 % habilitiert (18 Privatdozentinnen, 20 Professorinnen). In der vorliegenden Befragung waren 5,8 % Urologinnen habilitiert, was einer geringfügig höheren Quote entspricht. Saltzmann et al. zeigten in einer amerikanischen Studie aus dem Jahr 2016 vergleichbare Ergebnisse (Rate an Professorinnen in der Urologie: 5,6 %; [5]).

Nun könnte angenommen werden, dass die Feminisierung ein jüngerer Trend ist und der generelle strukturelle Wandel auch die Habilitationen erreichen wird. Jedoch spiegeln die Daten der jüngeren Zeit diesen Trend nicht wieder. So lag der Frauenanteil unter den der DGU gemeldeten Habilitant:innen in den letzten 5 Jahren bei 23,5 %, während die Frauenquote unter den Fachärzt:innen bereits bei 44 % lag.

Diese Daten zeigen, dass es einer Förderung von Habilitationen unter Urologinnen bedarf, wenn man eine Gleichstellung der Frauen erreichen möchte. Eine Habilitation gilt hierbei oft als Grundvoraussetzung für eine erfolgreiche wissenschaftliche Karriere und diese ist wiederum häufig an Führungspositionen geknüpft.

Betrachtet man die Literatur, so finden sich keine Hinweise auf Gestaltungsmöglichkeiten einer erfolgreichen Habilitationsförderung von Frauen. Ebenso existieren kaum belastbare Daten zur Forschungsförderung im Allgemeinen. Borgmann et al. konnten 2017 zeigen, dass durch Mentor:innen und Netzwerke geförderte Nachwuchswissenschaftler:innen vergleichsweise höhere Publikationsleistungen als ihre Kolleg:innen ohne solche strukturellen Voraussetzungen aufwiesen [6]. Die Aussagekraft der Befragung ist jedoch aufgrund der geringen Teilnehmerzahl von *n* = 78 begrenzt.

Mentoring- und Förderprogramme dienen neben dem inhaltlich-fachlichen Austausch der Stärkung des Anteils an Ärztinnen in Führungspositionen. Diese Programme sind mit positiven Ergebnissen verbunden. Dazu zählen eine optimierte Wissensvermittlung, Teamerfahrungen durch Unterstützung und Beratung und damit einhergehend ein positiver Gesamteffekt auf die berufliche Einstellung [7, 8].

Unsere Ergebnisse belegen den positiven Einfluss von Forschungsaufenthalten, Förderprogrammen, Forschungsfreistellungen und Mentoring-Programmen in der univariablen Betrachtung. Da naturgemäß diese Faktoren untereinander nicht unabhängig sind, erscheint die multivariable Analyse von besonderer Bedeutung zu sein. Anhand des Fragebogens konnten hier zwei unabhängige Prädiktoren für eine erfolgreiche Habilitation identifiziert werden: das Alter und ein Forschungsaufenthalt. Da eine Habilitation Zeit kostet, ist das Alter als Prädiktor nicht überraschend. Im Median, waren die 30 habilitierten Kolleginnen 40 (32 bis 63) Jahre alt, während das mediane Alter bei den Kolleginnen ohne Habilitation bei 37 (25 bis 67) Jahren lag. Als relevanter Prädiktor für eine erfolgreiche Habilitation konnte ein Forschungsaufenthalt identifiziert werden. Unter den 30 Kolleginnen mit Habilitation hatten 17 (57 %) einen Forschungsaufenthalt im In- oder Ausland absolviert.

Inwiefern die Forschungsaufenthalte durch ein Förder- oder Mentoring-Programm unterstützt worden waren und ggf. Einfluss auf den erfolgreichen Abschluss einer Habilitation hatten, wurde durch unsere Umfrage nicht erfasst. Insbesondere im Hinblick auf Forschungsförderung werden von der DGU verschiedene Förderprogramme angeboten. Hierzu zählt z. B. das „Ferdinand-Eisenberger-Stipendium“, das seit 2010 jährlich bis zu 5 Stipendiat:innen die Freistellung von der Klinik für ein Jahr ermöglicht, um ein definiertes Forschungsprojekt durchzuführen [9]. In diesem Programm wurden seit 2010 insgesamt 35 Stipendiat:innen gefördert. Der Frauenanteil betrug hier etwa 30 % (11/35). Zudem bietet das 2014 gegründete Netzwerk, GeSRU Academics, forschenden Nachwuchswissenschaftler:innen in der Urologie eine Plattform zu Vernetzung, zum wissenschaftlichen Austausch und zur Förderung individueller Forschungsfähigkeiten [6]. Auf europäischer Ebene werden Nachwuchswissenschaftler:innen durch die European Association of Urology (EAU) seit 2011 durch die Young Academic Urologists (YAU) vernetzt, mit dem Ziel der Förderung von hochqualitativer Forschung. Ein speziell auf Urologinnen ausgerichtetes Förderprogramm existiert bisher noch nicht.

Die Ergebnisse unserer Befragung zeigen, dass sich eine effektive wissenschaftliche Förderung von Urologinnen auf die Unterstützung von Forschungsaufenthalten konzentrieren sollte. Eine alleinige Freistellung im Sinne von „Zeit für Forschung“ scheint nicht zielführend zu sein. Ein Mentoring sollte unterstützend angeboten werden.

Verschiedene Limitationen müssen erwähnt werden. Insbesondere die Anzahl der habilitierten Teilnehmerinnen war gering, was die statistische Auswertung erschwert und die Aussagekraft insbesondere der multivariablen Analyse reduziert. Zudem wurde die Umfrage nur an weibliche DGU-Mitglieder geschickt, wodurch ein Selektionsbias entstanden sein kann. Trotz dieser Limitationen ist zu bemerken, dass dies die erste umfassende Analyse zu Einflussfaktoren auf eine erfolgreiche Habilitation unter Urologinnen ist.

## Schlussfolgerung

Die Ergebnisse der vorliegenden Umfrage ermöglichen erstmals eine genaue Analyse der aktuellen Situation von Ärztinnen in der DGU. Neben zahlreichen weiteren Aspekten, wie der Zufriedenheit mit der beruflichen Situation oder der Vereinbarkeit von Familie und Beruf, konnten durch die Befragung wichtige Themen für die wissenschaftliche Karriere von Frauen in der Urologie erfasst werden. Eine Förderung von Habilitationen unter Frauen erscheint erstrebenswert, um den Frauenanteil in Führungspositionen zu steigern. Die Ergebnisse der Datenauswertung zeigen, dass insbesondere die Förderung von Forschungsaufenthalten explizit für Frauen ein vielversprechender Ansatz ist. Eine weitere umfassende Analyse der Gründe für die geringe Habilitationsrate unter Frauen ist erforderlich, um gezielte Fördermöglichkeiten zu identifizieren und zu schaffen und so mehr Frauen in Führungspositionen zu bringen.

## Fazit für die Praxis

- Der Anteil an Ärztinnen im Fachgebiet Urologie nimmt stetig zu. In Führungspositionen ist diese Entwicklung jedoch noch nicht angekommen.
- Die AG Urologinnen hat sich eine strukturierte Förderung von Ärztinnen in der Urologie zum Ziel gemacht.
- Hierfür erfolgte Anfang 2022 eine Online-Befragung der weiblichen DGU-Mitglieder.
- Eine effektive wissenschaftliche Förderung von Frauen scheint insbesondere durch Forschungsaufenthalte bzw. Forschungsangebote möglich zu sein.
- Hierfür sollte ein speziell auf Frauen ausgerichtetes Förderprogramm angeboten werden.
